# The Impact of Caffeine Intake on Patients with Systemic Lupus Erythematosus: Protect Yourself, Drink More Coffee!

**DOI:** 10.31138/mjr.31.4.374

**Published:** 2020-12-28

**Authors:** Valeria Orefice, Fulvia Ceccarelli, Cristiana Barbati, Carlo Perricone, Cristiano Alessandri, Fabrizio Conti

**Affiliations:** 1 Lupus Clinic, Dipartimento di Scienze Cliniche, Internistiche, Anestesiologiche e Cardiovascolari, Sapienza Università di Roma, Rome, Italy; 2 Reumatologia, Dipartimento di Medicina e Chirurgia, Università di Perugia, Perugia, Italy

Systemic lupus erythematosus (SLE) is an autoimmune disease mainly affecting mostly young women, potentially involving any organ/system. The central role of environmental factors in disease pathogenesis has been widely demonstrated: among these, an emerging interest has been pointed to dietary factors.^1–4^ In this context, the spectrum of research on caffeine, one of the most widely consumed products in the world, is exponentially growing during the last decade. Indeed, caffeine, acting as a non-specific phosphodiesterase inhibitor, seems to be able to interact with multiple components of the immune system, influencing both innate and adaptive response.^5–8^

Recently, we performed a study to evaluate the impact of caffeine intake on SLE activity and phenotype.^9^ By analysing a large monocentric cohort, we identified an inverse correlation between caffeine consumption and disease activity, in terms of SLEDAI-2k values and serum cytokines levels.^9^ Our results suggest that a moderate caffeine intake could modulate disease activity, and thus influence chronic damage. Indeed, we were able to demonstrate that lower caffeine intake was associated with more frequent major organ involvement - such as renal and neuropsychiatric manifestations - and anti-dsDNA positivity.

Our results are concordant with a previous published study evaluating a Colombian SLE cohort, in which coffee consumption was positively associated with 6 months clinical remission.^10^ Moreover, Kiyohara and colleagues analysed the association between coffee intake and SLE risk, demonstrating only a marginal dose-dependent association.^11^

Several studies were published so far about the contribution of diet in SLE aetiopathogenesis, suggesting a possible influence on systemic inflammatory status, leading to modifications on inflammatory cell activity and cytokine levels.^12^ Taken together, data available from the literature suggest that a diet rich in vitamin D and A, polyunsaturated fatty acids, and phenols could be able to reduce the inflammatory burden.^12^ Furthermore, a protective role of the traditional Mediterranean diet and a low-sodium dietary regimen has been recently suggested.^13^ We added new information about the relationship between caffeine and SLE; in fact, despite the worldwide spread of caffeine usage, very few data on SLE have been published so far.

Furthermore, our study provided deeper evidence on the role of caffeine on disease activity, by identifying an inverse correlation between daily caffeine intake and inflammatory cytokine levels. Indeed, for the first time, we demonstrated significantly lower serum levels of IFNγ, IFNα, IL-17, and IL-6 in SLE patients with higher daily caffeine intake.^9^ In this regard, Iris and colleagues demonstrated that *in vitro* dose-dependent treatment with caffeine could downregulate mRNA levels of key inflammation-related genes in peripheral blood mononuclear cells of healthy donors, and similarly, decrease levels of different inflammatory cytokines in a dose-dependent way.^14^

In conclusion, in the last years, a growing interest has focused on the role of caffeine in the immune-related diseases pathogenesis and phenotype. In particular, *in vitro* and *in vivo* studies suggest a possible immunoregulatory dose-dependent effect exerted through the modulation of several serum cytokine levels; in the same way, caffeine could influence SLE phenotype favouring less severe disease manifestations.
